# Association of classical markers and establishment of the dyslipidemic sub-phenotype of sickle cell anemia

**DOI:** 10.1186/s12944-017-0454-1

**Published:** 2017-04-11

**Authors:** Milena Magalhães Aleluia, Caroline Conceição da Guarda, Rayra Pereira Santiago, Teresa Cristina Cardoso Fonseca, Fábia Idalina Neves, Regiana Quinto de Souza, Larissa Alves Farias, Felipe Araújo Pimenta, Luciana Magalhães Fiuza, Thassila Nogueira Pitanga, Júnia Raquel Dutra Ferreira, Elisângela Vitória Adorno, Bruno Antônio Veloso Cerqueira, Marilda de Souza Gonçalves

**Affiliations:** 1 Laboratório de Hematologia e Genética Computacional, Instituto Gonçalo Moniz - IGM, Rua Waldemar Falcão, 121, Candeal - Salvador/BA, CEP: 40296-710 Bahia Brazil; 2 Universidade Federal da Bahia (UFBA), Salvador, Bahia Brazil; 3 Centro de Referência a Doença Falciforme de Itabuna, Itabuna, Bahia Brazil; 4 Universidade Estadual de Santa Cruz (UESC), Ilhéus, Bahia Brazil; 5 Universidade Estadual da Bahia (UNEB), Salvador, Bahia Brazil

**Keywords:** Sub-phenotype, Sickle cell anemia, Dyslipidemia, α-thalassemia

## Abstract

**Background:**

Sickle cell anemia (SCA) patients exhibit sub-phenotypes associated to hemolysis and vaso-occlusion. The disease has a chronic inflammatory nature that has been also associated to alterations in the lipid profile. This study aims to analyze hematological and biochemical parameters to provide knowledge about the SCA sub-phenotypes previously described and suggest a dyslipidemic sub-phenotype.

**Methods:**

A cross-sectional study was conducted from 2013 to 2014, and 99 SCA patients in steady state were enrolled. We assessed correlations and associations with hematological and biochemical data and investigated the co-inheritance of -α^3.7Kb^-thalassemia (-α^3.7Kb^-thal). Correlation analyses were performed using Spearman and Pearson coefficient. The median of quantitative variables between two groups was compared using *t*-test and Mann-Whitney. *P*-values <0.05 were considered statistically significant.

**Results:**

We found significant association of high lactate dehydrogenase levels with decreased red blood cell count and hematocrit as well as high levels of total and indirect bilirubin. SCA patients with low nitric oxide metabolites had high total cholesterol, high-density lipoprotein cholesterol, and low-density lipoprotein cholesterol and reduced very low-density cholesterol, triglycerides, direct bilirubin level and reticulocyte counts. In SCA patients with high-density lipoprotein cholesterol greater than 40 mg/dL, we observed increased red blood cell count, hemoglobin, hematocrit, and fetal hemoglobin and decreased nitric oxide metabolites levels. The presence of -α^3.7Kb^-thal was associated with high red blood cell count and low mean corpuscular volume, mean corpuscular hemoglobin, platelet count and total and indirect bilirubin levels.

**Conclusions:**

Our results provide additional information about the association between biomarkers and co-inheritance of -α^3.7Kb^-thal in SCA, and suggest the role of dyslipidemia and nitric oxide metabolites in the characterization of this sub-phenotype.

## Background

Sickle cell anemia (SCA) represents the homozygous condition of the beta S (β^S^) globin allele, is the more severe genotype of sickle cell disease (SCD). SCA is characterized by hemolysis, chronic and acute inflammation, vaso-occlusive complications, multiple organ damage, and reduced patient survival [1]. The pathophysiology of SCA is complex and influenced by hypoxia, acidosis and cell dehydration, which contribute to the HbS polymerization that leads to erythrocyte deformation [2]. The changes in the blood flow may contribute to increase oxidative stress, leading to vaso-occlusive episodes (VOE) complications, such as acute chest syndrome (ACS), stroke, priapism, gallstone, and retinopathy [3, 4].

SCA patients exhibit sub-phenotypes that very often overlap, although they are helpful to understand the pathophysiological mechanisms of the disease. The vaso-occlusive sub-phenotype has been associated with blood viscosity and VOE, and the hemolytic sub-phenotype has been related with hemolysis and endothelial dysfunction, with alterations in nitric oxide (NO) and lactate dehydrogenase (LDH) levels, as well as hematological parameters [5].

Hemolysis in the SCD exhibits heterogeneous intensities due to changes in the hemoglobin (Hb) concentration altering laboratory biomarkers, such as reticulocyte and red blood cells (RBC) counts, bilirubin and its fractions levels, lactate dehydrogenase (LDH) and mean corpuscular volume (MCV) [5]. The NO is a potent vasodilator and is an important endothelial mediator in the natural control of vascular tone, adhesion, platelet aggregation and thrombosis [6].

Lipid metabolism in SCD is potentially associated with hemolytic profile and endothelial dysfunction. Similarly, dyslipidemia has been described among patients with different SCD genotypes, with increased low-density lipoprotein cholesterol (LDL-C) and decreased high-density lipoprotein cholesterol (HDL-C) levels, revealing a potential predictor biomarker of disease severity [7, 8]. The HDL-C plays important role in reducing the risk of hemolysis and improving endothelial dysfunction and contributes to a better clinical outcome. HDL-C fractions, such as pro-HDL-C, have been associated with inflammatory disease [9]. The very low-density cholesterol (VLDL-C) fraction and triglycerides increase the number of LDL-C receptors and its systemic levels, contributing to the dyslipidemic sub-phenotype in SCD [8]. This sub-phenotype explained the high oxidative stress secondary to the intravascular hemolysis in SCD patients. However, there are several reports about low HDL-C [7, 8] and increased triglycerides [5, 10, 11] in SCD patients, features widely recognized in the general population as important factors in cardiovascular disease [12].

Since several studies have identified the lipids as important biomarkers for cardiovascular disease [12], thrombosis and SCD dyslipidemia [7, 8], we hypothesize that assessing laboratory measurement of the lipid profile may aid the clinical management of SCA patients.

This study aims to analyze hematological and biochemical parameters to provide knowledge about the SCA sub-phenotypes previously described and suggest a dyslipidemic sub-phenotype to SCA.

## Methods

### Subjects

We developed a cross-sectional study conducted from 2013 to 2014, and 99 SCA patients in steady state were included. Patients had not received blood transfusion at least six months before the blood collection, and they were not treated with hydroxyurea. The present study received approval from the institutional review board of the Gonçalo Moniz Institute at the Oswaldo Cruz Foundation (IGM-FIOCRUZ – Bahia - Brazil) and is in compliance with the guidelines for human research established by the Declaration of Helsinki, as well as its subsequent revisions. Informed written consent was obtained from all study subjects or their guardians who agreed to participate in the study and authorized the use of collected samples. SCA patients were seen at Sickle Cell Disease Reference Center of Itabuna, Itabuna, Bahia, Brazil.

### Laboratory methods

Hematological analyses were performed using the Sysmex KX-21 N™ Automated Hematology Analyzer (Sysmex Corporation, Tokyo, Japan), and biochemical analyses were performed using the Cobas Automated Analyzer (Roche Diagnostics, Salt Lake city, Utah, USA). We investigated the hemoglobin profile and the fetal hemoglobin (HbF) concentration by high-performance liquid chromatography using a hemoglobin testing system (HPLC/Variant-I; Biorad, Hercules, CA, USA). NO metabolites (NOm) were determined in serum by Griess reaction, as previously described [13]. Genomic DNA was extracted from peripheral blood using a QIAamp DNA Blood Mini Kit (QIAGEN, Hilden, Vestfália, Germany) in accord to manufacturer’s recommendations. In addition, -α^3.7Kb^-thal detection was investigated by allele-specific polymerase chain reaction (PCR) [14]. Blood samples were analyzed at the Laboratory of Hematology, Genetic and Computational Biology (LHGB-IGM-FIOCRUZ) and at the College of Pharmaceutical Sciences (UFBA).

### Statistical analysis

The variables selected were expressed as the means, medians and percentile. Distribution of quantitative variables was analyzed using the Shapiro-Wilk test. The median of quantitative variables between two groups was compared using *t*-test for data with normal distribution and Mann-Whitney for nonparametric data.

In order to perform the analysis of laboratory parameters, we decided to use the median value for LDH and NOm. Thus, we divided the patients into two groups: low LDH (less than 1094.0 U/L) and high LDH (at least 1094.0 U/L); and low NOm (less than 35.75 μM) and high NOm (at least 35.75 μM). HDL-C level of 40 mg/dl is reported to be the normal reference range limit and we divided the groups into low HDL-C (less than 40 mg/dl) and high HDL-C (at least 40 mg/dl). The presence of -α^3.7Kb^-thal co-inheritance was also associated with laboratory data in SCA patients.

Correlation analyses were performed between variables using Spearman and Pearson coefficient (R). Data were tabulated and analyzed using the Statistical Package for Social Sciences (SPSS) version 20.0 (IBM, New York, NY). JMP software was used to assemble the correlation graphs, P-values <0.05 were considered statistically significant.

## Results

### Association of LDH with hemolysis biomarkers

The low LDH group (less than 1094.0 U/L) included 29 SCA patients within the interval of 553.50 to 747.00 U/L with a mean of 644.97 U/L. The high LDH group (greater than or equal to 1094.0 U/L) included 70 SCA patients within the interval ranging from 1036.50 to 1885.25 U/L with a mean of 1466.60 U/L.

The high LDH group showed decreased values of RBC count (*p* = 0.015), hemoglobin (Hb) (*p* = 0.040) and hematocrit (Ht) (*p* = 0.016) concentration and increased levels of total (*p* = 0.028) and indirect bilirubin (*p* = 0.025), mean corpuscular volume (MCV) (*p* = 0.030) and mean corpuscular hemoglobin (MCH) (*p* = 0.032) concentrations (Table 1). An increase in the monocyte count was also observed (*p* = 0.017) (Table 1).

**Table 1:** Association of laboratory data and LDH, NOm and HDL-C levels in SCA patients

	LDH <1094.0 U/L (*N* = 29)	LDH ≥1094.0 U/L (*N* = 70)	Nitric Oxide m <35.75 μM (*N* = 59)	Nitric Oxide m ≥35.75 μM (*N* = 38)	HDL-C <40.00 mg/dL (*N* = 81)	HDL-C ≥40.00 mg/dL (*N* = 18)	P1	P2	P3
	Median (25^th^ -75^th^)	Median (25^th^ -75^th^)	Median (25^th^ -75^th^)	Median (25^th^ -75^th^)	Median (25^th^ -75^th^)	Median (25^th^ -75^th^)	*p*-value		
Hemolysis									
RBC, x10^12^/L	2.7 (2.4–3.1)	2.5 (2.2–2.7)	2.6 (2.3–2.8)	2.4 (2.2–2.8)	2.5 (2.2–2.8)	2.7 (2.4–3.1)	0.015*	0.176*	**0.018**
Hemoglobin, g/dL	7.9 (7.3 –9.1)	7.5 (7.0–8.5)	7.6 (7.20–8.5)	7.5 (6.6–8.5)	7.6 (7.1–8.5)	7.9 (7.1–10.0)	0.040*	0.349	**0.049**
Hematocrit, %	23.1 (21.3–26.7)	21.5 (19.8–24.0)	21.7 (20.20–25.5)	21.8 (18.9–24.1)	21.7 (20.0–24.1)	22.9 (20.8–27.7)	0.016*	0.346	**0.036**
MCV, fL	83.8 (78.8–88.1)	88.1 (83.5–92.8)	85.1 (81.20–89.8)	88.2 (83.0–94.6)	86.4 (81.6–91.3)	85.0 (80.9–90.5)	**0.030**	0.120	0.403
MCH, fL	29.1 (26.5–31.3)	30.5 (28.7–32.6)	29.9 (27.80–32.1)	30.7 (27.8–32.7)	30.4 (28.0–32.7)	29.6 (27.7–31.3)	**0.032**	0.260	0.288
Total bilirubin, mg/dL	1.9 (1.3–3.4)	3.1 (1.8–3.9)	2.3 (1.60 –3.8)	3.2 (1.8–3.9)	2.8 (1.8–3.8)	2.0 (0.5–3.8)	**0.028**	0.183	0.983*
Direct bilirubin, mg/dL	0.4 (0.3–0.5)	0.5 (0.3–0.6)	0.4 (0.30–0.5)	0.5 (0.4–0.7)	0.5 (0.3–0.6)	0.4 (0.2–0.6)	0.166	**0.024**	0.501
Indirect bilirubin, mg/dL	1.5 (0.9–2.6)	2.4 (1.4–3.3)	2.0 (1.20–3.3)	2.4 (1.4–3.3)	2.2 (1.3–3.2)	1.4 (0.4–3.2)	**0.025**	0.085*	0.958*
LDH, U/L			1096.0 (837.0–1713.5)	1201.0 (830.0–1931.2)	1096.0 (837.0 –1713.5)	937.5 (650.0–1546.7)		0.152*	0.416*
Reticulocyte count	5.9 (3.9–8.2)	5.25 (4.40–8.12)	5.0 (3.9–7.1)	7.1 (4.9–8.6)	5.3 (4.2–7.9)	5.9 (4.0–10.7)	0.775*	**0.012**	0.498*
Hemoglobin profile									
Fetal hemoglobin, %	11.4 (4.6–17.4)	8.7 (5.2–13.0)	9.6 (5.3–14.8)	8.9 (3.8–13.0)	7.9 (4.2–13.1)	13.4 (8.7–19.0)	0.077*	0.311*	**0.005**
NO metabolites									
NOm, μM	34.6 (29.1–42.8)	36.1 (27.9–51.0)			37.6 (29.1–51.1)	30.5 (26.3–36.8)	0.702		**0.024**
Leukocytes									
WBC, x 10^9^/L	14.3 (11.5–16.6)	13.7 (11.1–15.8)	13.7 (11.1–16.0)	13.90 (11.7–16.6)	13.9 (11.4–15.9)	12.2 (10.4–16.9)	0.508	0.450	0.751*
Neutrophil, x 10^9^/L	6664.0 (4209.0–8355.0)	5382.0 (4080.0–7113.5)	5424.0 (3892.0–7182.0)	6088.0 (4485.2–7203.5)	5760.0 (4210.5–71.82.0)	5516.0 (3361.5–7691.0)	0.305	0.387	0.824
Eosinophil, x 10^9^/L	714.0 (255.5–1967.0)	685.0 (288.7–1608.7)	800.0 (291.0–1807.0)	704.5 (281.5–2109.0)	805.0 (303.5–1925.0)	502.0 (238.5–1328.2)	0.319*	0.336*	0.415*
Lymphocyte, x 10^9^/L	5408.0 (4540.0–6726.5)	5580.0 (4261.0–7564.5)	5408.0 (3990.0–7224.0)	5939.5 (4491.5–7569.7)	5560.0 (4359.0–7209.5)	5798.0 (3751.5–7728.0)	0.508	0.439*	0.647
Monocyte, x 10^9^/L	290.0 (169.0–419.0)	454.5 (248.5–763.2)	338.0 (222.0–576.0)	377.5 (212.5–863.0)	330.0 (212.0–680.5)	492.0 (309.0–720.7)	0.017*	0.296*	0.792
Platelets									
Platelet, x10^3^/mL	450.0 (350.0–514.0)	439.0 (367.7–550.2)	445.0 (384.0–558.0)	419.5 (342.5–514.5)	442.0 (358.0–559.0)	439.5 (402.0–497.0)	0.982	0.337	0.345
Lipid profile									
Total Cholesterol, mg/dL	132.0 (85.0–152.0)	120.5 (101.5–144.5)	131.0 (111.0–153.0)	109.0 (85.5–146.0)			0.933	0.014*	
HDL-C, mg/dL	33.0 (28.5–40.5)	31.0 (26.0–36.2)	34.0 (28.0–40.0)	30.0 (26.0–33.2)			0.108	**0.018**	
LDL-C, mg/dL	66.0 (41.5–87.5)	67.0 (44.0–88.2)	74.0 (57.0–99.0)	51.5 (30.5–84.2)	66.0 (40.0–84.0)	92.0 (73.5–128.2)	0.982	0.007*	**<0.001**
VLDL-C, mg/dL	22.0 (16.0–33.0)	22.0 (17.0–31.0)	21.0 (16.0–26.0)	28.0 (18.0–35.0)	23.0 (17.0–32.0)	20.0 (15.5–28.5)	0.948	0.003*	**0.036**
Triglycerides, mg/dL	109.0 (81.0–164.0)	110.0 (84.0–155.2)	103.0 (78.0–130.0)	139.0 (91.7–176.5)	113.0 (83.5–158.5)	101.5 (78.7–144.0)	0.951	0.008*	0.441

*RBC* red blood cell, *MCV* mean cell volume, *MCH*: mean corpuscular hemoglobin, *LDH* dehydrogenase, *NOm*, nitric oxide metabolites, *WBC* white blood cell, *HDL-C* high-density lipoprotein cholesterol, *LDL-C* low-density lipoprotein cholesterol, *VLDL-C* very low-density lipoprotein cholesterol. Bold values indicate significance at p < 0.05; p-value obtained using Mann-Whitney test. *p-value obtained using *t*-test. P1 = LDH <1094.0 U/L X LDH ≥ 1094.0 U/L; P2 = NOm < 35.75 μM X NOm ≥ 35.75 μM; P3 = HDL-C < 40.0 mg/dl x HDL-C ≥40.0 mg/dL

**Fig. 1:**
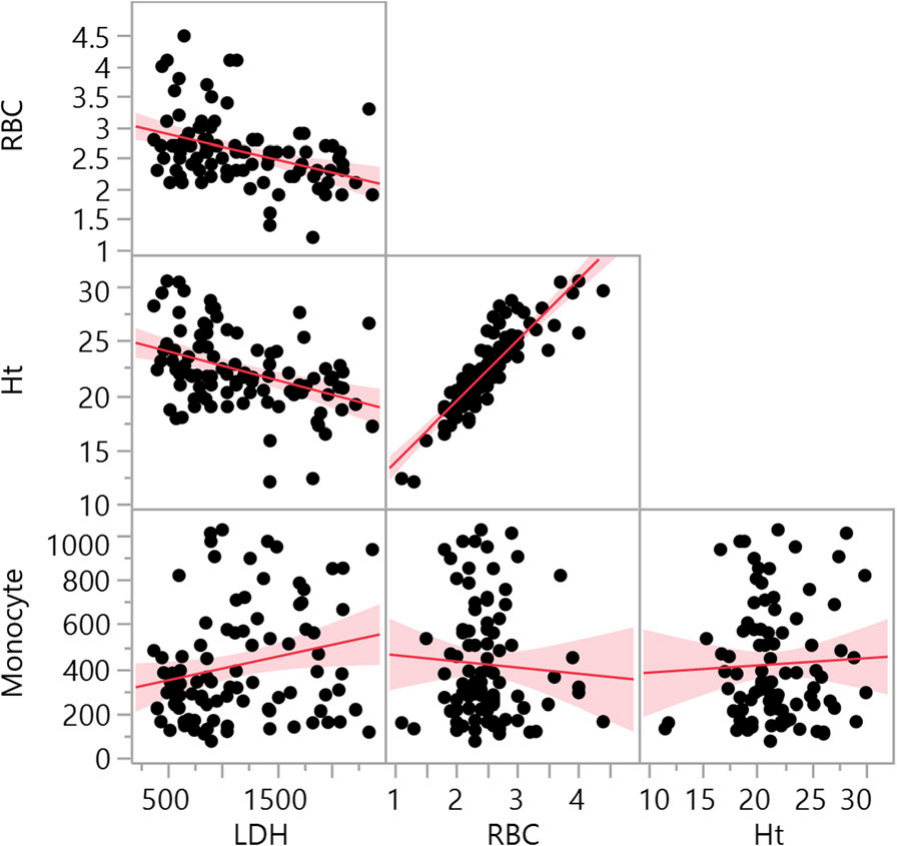
Correlation of laboratory data between LDH levels in SCA. Legend: RBC count (*R* = -0.3906; *p* <0.001); hematocrit (Ht) (*R* = -0.4006; *p* <0.001); monocyte count (*R* = 0.2205; *p* <0.034)

The LDH was negatively correlated to RBC count (*R* = -0.3906; *p* <0.001) and Ht (*R* = -0.4006; *p* <0.001), and positively correlated to monocytes count (*R* = 0.2205, *p* = 0.034) (Fig. 1).

### Association of NOm with lipids biomarkers

The group with low levels of NOm (less than 35.75 μM) includes 59 SCA patients, with an interval of 29.55 to 34.56 μM and a mean of 28.88 μM. The group with high levels of NOm (greater than or equal to 35.75 μM) included 38 SCA patients with an interval of 43.06 to 66.49 μM and a mean of 58.62 μM.

The group with high levels of NOm was significantly associated with an increased reticulocyte count (*p* = 0.012), direct bilirubin (*p* = 0.024), VLDL-C (*p* = 0.003) and triglycerides levels (*p* = 0.008). In the same group, we observed decreased total cholesterol (*p* = 0.014), HDL-C (*p* = 0.018) and LDL-C levels (*p* = 0.007).

**Fig. 2:**
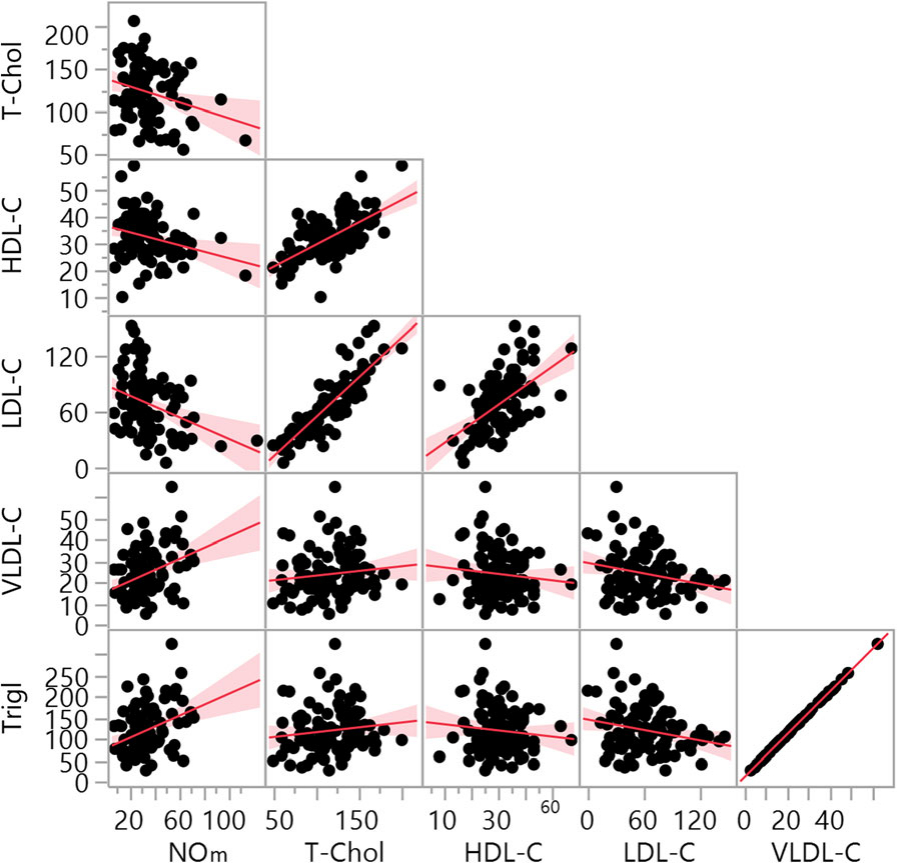
Correlation of laboratory data between NOm levels in SCA. Legend: Total cholesterol (T-Chol) (*R* = -0.2650; *p* = 0.010); HDL-C (*R* = -0.2693; *p* = 0.008); LDL-C (*R* = -0.3481; *p* <0.001); VLDL-C (*R* = 0.3614; *p* <0.001) and triglycerides (Trigl) (*R* = 0.3586; *p* <0.001)

Correlation analyses established from the measurement of NOm were negatively correlated with total cholesterol (*R* = -0.2650, *p* = 0.010), HDL-C (*R* = -0.2693, *p* = 0.008) and LDL-C (*R* = -0.3481; *p* <0.001). NOm was also positively correlated with VLDL-C (*R* = 0.3614; *p* <0.001) and triglycerides (*R* = 0.3586; *p* <0.001) (Fig. 2).

### Association of HDL-C with hematologic and NOm biomarkers

The low HDL-C group (less than 40.00 mg/dL) includes 81 SCA patients with an interval of 26.00–34.00 mg/dL and a mean of 29.65 mg/dL. The high HDL-C group (greater than or equal to 40.00 mg/dL) includes 18 SCA patients with an interval of 41.00–45.00 mg/dL and a mean of 44.11 mg/dL.

HDL-C concentrations greater than 40 mg/dL were associated with increased RBC count (*p* = 0.018), Hb (*p* = 0.049), Ht concentration (*p* = 0.036), HbF (p = 0.005) and LDL-C levels (*p* <0.001) (Table 1). Likewise, we observed a decreased NOm (*p* = 0.024) and VLDL-C levels (*p* = 0.036) (Table 1).

**Fig. 3:**
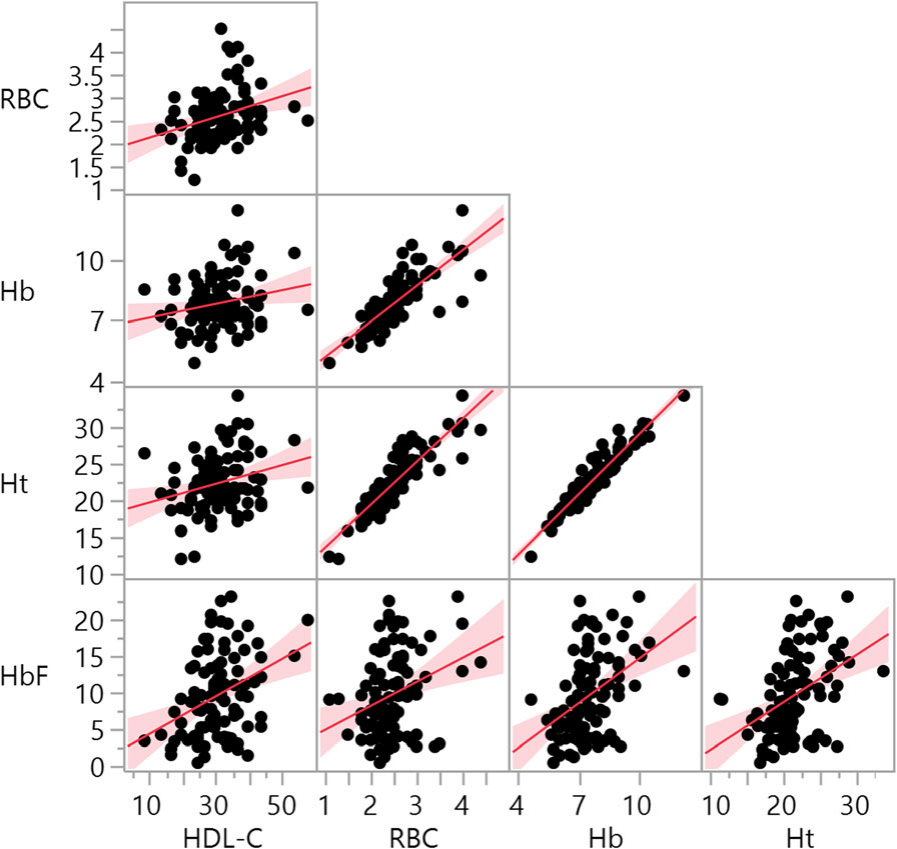
Correlation of laboratory data between HDL-C levels in SCA. Legend: RBC count (*R* = 0.3041; *p* = 0.002); hemoglobin (Hb) (*R* = 0.2139; *p* = 0.036); hematocrit (Ht) (*R* = 0.2706; *p* = 0.007) and fetal hemoglobin (HbF) (*R* = 0.3652; *p* <0.001)

HDL-C was positively correlated with RBC count (*R* = 0.3041, *p* = 0.002), Hb (*R* = 0.2139, *p* = 0.036), Ht (*R* = 0.02706; *p* = 0.007) and HbF (*R* = 0.3652; *p* <0.001) (Fig. 3).

### Detection of with -α^3.7Kb^-thalassemia

We identified 85 SCA patients non-carriers of -α^3.7Kb^-thal and 14 patient carriers of the deletion, 11 heterozygous (-/αα) and 3 homozygous (-α/-α). The group with -α^3.7Kb^-thal exhibited high RBC counts (*p* = 0.012) and low MCV (*p* <0.001) and MCH (*p* <0.001). This group also had low levels of total (*p* = 0.026) and indirect (*p* = 0.026) bilirubin. Reduced lymphocyte (*p* = 0.036) and platelets count (*p* = 0.003) (Table 2) were also observed.

**Table 2:** Association of laboratory data between SCA patients in the presence or absence of -α^3.7Kb^-thalassemia

	Presence of -α^3.7Kb^ -thalassemia (*N* = 14)	Absence of -α^3.7Kb^ -thalassemia (*N* = 85)	P1
	Median (25^th^ -75^th^)	Median (25^th^ -75^th^)	*p*-value
Hemolysis			
RBC, x10^12^/L	2.9 (2.5–3.3)	2.5 (2.2–2.7)	**0.012**
Hemoglobin, g/dL	8.2 (7.3–8.8)	7.6 (7.1–8.3)	0.814*
Hematocrit, %	23.8 (21.4–25.5)	21.7 (19.9–24.1)	0.839*
MCV, fL	80.3 (76.3–83.0)	88.0 (83.6–93.2)	**0.001**
MCH, fl	27.7 (25.7–29.0)	30.8 (28.8–32.7)	**0.001**
Total bilirubin, mg/dL	1.8 (1.2–2.7)	2.8 (1.7–3.9)	**0.026**
Direct bilirubin, mg/dL	0.4 (0.2–0.5)	0.5 (0.3–0.6)	0.180
Indirect bilirubin, mg/dL	1.3 (0.9–2.2)	2.3 (1.3–3.4)	**0.026**
LDH, U/L	958.0 (852.2–1845.7)	1120.0 (762.5–1693.0)	0.848*
Reticulocyte count	4.6 (3.7–8.6)	5.8 (4.3–8.1)	0.736*
Hemoglobin profile			
Fetal hemoglobin, %	9.4 (3.4–13.9)	9.3 (5.2–14.4)	0.651
NO metabolites			
NOm, μM	33.9 (20.7–44.4)	35.7 (28.8–48.0)	0.400
Leukocytes			
WBC, x 10^9^/L	12.3 (7.6–14.8)	14.0 (11.2–16.1)	0.161
Neutrophil, x 10^9^/L	5124.0 (3830.2–7061.7)	5796.0 (4208.5–7225.0)	0.550
Eosinophil, x 10^9^/L	582.5 (232.5–1936.5)	800.0 (303.50–1785.5)	0.676*
Lymphocyte, x 10^9^/L	3711.0 (2695.5–6960.7)	5688.0 (4533.0–7269.0)	**0.036**
Monocyte, x 10^9^/L	202.0 (124.5–432.7)	380.0 (252.0–702.0)	0.120*
Platelets			
Platelet, x10^3^/mL	335.5 (239.2–443.0)	450.0 (382.0–559.0)	**0.003**
Lipid profile			
Total Cholesterol, mg/dL	118.5 (106.7–142.2)	123.0 (97.5–148.0)	0.810
HDL-C, mg/dL	30.0 (24.7–38.0)	32.0 (27.0–37.5)	0.376
LDL-C, mg/dL	68.0 (56.7–99.5)	67.0 (41.0–87.0)	0.396
VLDL-C, mg/dL	22.5 (18.0–28.7)	22.0 (16.5–32.0)	0.936
Triglycerides, mg/dL	111.0 (89.5–142.5)	110.0 (82.5–158.5)	0.928

*RBC* red blood cells, *MCV* mean cell volume, *MCH* mean corpuscular hemoglobin, *LDH* lactate dehydrogenase, *NOm* nitric oxide metabolites, *WBC* white blood cell, *HDL-C* high-density lipoprotein cholesterol, *LDL-C* low-density lipoprotein cholesterol, *VLDL-C* very low-density lipoprotein cholesterol. Bold values indicate significance at *p* <0.05; p-value obtained using Mann- Whitney. *p-value obtained using *t*-test. P1 = presence of -α^3.7Kb^-thalassemia X absence of -α^3.7Kb^-thalassemia

## Discussion

Our study was designed to evaluate laboratory data and their associations with LDH, NOm and HDL-C levels as well as the co-inheritance of -α^3.7Kb^-thal in steady state SCA patients. We identified associations among the biomarkers related to hemolysis, vaso-occlusion, endothelial dysfunction, inflammation and lipid metabolism. Based in the knowledge about the SCA sub-phenotypes previously described and our results, we suggest the inclusion of the dyslipidemic sub-phenotype.

The LDH is a classical biomarker of intravascular hemolysis, and RBC disruption results in the simultaneous release of Hb, heme and arginase into blood stream [5]. We observed association of elevated LDH levels with other classical hemolysis biomarkers, such as decreased RBC count, Hb, Ht, as well as with increased MCV, MCH, total and indirect bilirubin and monocyte count, suggesting that our data are consistent with the findings commonly described in the literature [15]. In addition, the elevated monocyte count may be associated to hemolysis due to increased phagocytic activity and removal of excess lysed RBC from the peripheral blood [16].

Our evaluation of NOm between the groups showed in the low NOm group reduced levels of direct bilirubin, VLDL-C and triglycerides as well as reticulocyte count. We also observed in the same group increased total cholesterol, HDL-C and LDL-C levels. NO biological properties, such as increasing vascular permeability, inhibition of platelet aggregation and endothelial activation, play important role in SCA [17]. However, the release of Hb and arginase from the RBC limits NO bioavailability [5, 17, 18] and promotes a vaso-constrictor status. It is known that bilirubin can exert antioxidant properties *in vitro*, acting as an endogenous scavenger of both NO and reactive nitrogen species [19]. Thus, it is possible that the decreased NOm levels observed may be due to the potential scavenging activities of bilirubin.

Importantly, SCA patients exhibit a dyslipidemic phenotype, as previously described [7, 8]. The observed decreased levels of total cholesterol, HDL-C and LDL-C and increased VLDL-C and triglycerides are not new findings; however, the dyslipidemic characteristic has not been previously associated to NOm levels. Altered serum lipid levels have been associated to endothelial dysfunction and as a risk factor for pulmonary hypertension [20]. In addition, it was identified a pro-inflammatory fraction of HDL (pro-HDL), which has been increased and also contribute to the pathophysiology of pulmonary vascular disease in SCD patients [21]. Thus, our results reinforce the presence of a dyslipidemic sub-phenotype in SCA, as well as the previous association with vascular alterations.

In the group of patients where HDL-C levels were greater than 40.0 mg/dL we identified an improvement of the hematological features, since we observed high RBC count, hemoglobin and hematocrit levels. These results are in accord to other [8] that also described association between HDL-C levels and hematologic parameters. Thus, HDL-C plays an important role as a prognostic marker in SCA. We also found an association between HbF and HDL-C levels. The HbF plays an important role in the modulation of SCA pathogenesis, and its levels are generally inversely related to the severity of SCA for particular sub-phenotype. Therefore, the increase in HbF levels reduces HbS polymerization and, consequently, VOE, pain crisis and hospitalization [2, 22]. HDL-C exhibits anti-inflammatory, antioxidant, platelet anti-aggregation, anticoagulant and pro-fibrinolysis activity [23]. In patients with SCA, high HDL-C levels may promote a reduction in the risk of intravascular hemolysis and endothelial injury [8].

Regarding the co-inheritance of α-^3.7Kb-^thal, we observed a frequency of 0.14 of the deletion. The coexistence of -α^3.7Kb^-thal in SCA patients is associated with the improvement of anemia, as suggested by increased RBC counts and decrease of total and indirect bilirubin levels [5]. In addition, we also observed decrease of MCV and MCH in accordance with previous report [24]. Individuals with co-inheritance of α-thal have a low count of dense cells probably due to reduced RBC mechanical fragility. We also verified reduced lymphocyte counts, consistent with previous report [24].

The α-thal co-inheritance also exhibited a significant reduction in platelet count. Platelets are essential for hemostasis but may contribute to the inflammatory process [25]. To date, we have not found studies that show the influence of α-thal on platelet count in SCA patients [26].

Previous studies have shown that SCA patients exhibit some distinct sub-phenotypes, which often overlap. These sub-phenotypes are identified based on laboratory evaluation, α-thal co-inheritance and clinical history [5, 27]. The role of lipids in the inflammatory response has been suggested, due to anti-inflammatory properties of HDL-C [12] and pro-inflammatory properties of LDL-C [9]. Thus, considering that SCA patients have a chronic inflammatory status, the assessment of the lipid profile and the study of the dyslipidemic sub-phenotype may be helpful to improve the knowledge about the heterogeneous clinical manifestations.

**Fig. 4:**
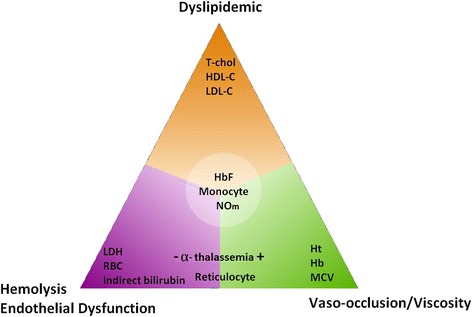
Model of dyslipidemic, hemolysis and endothelial dysfunction, vaso-occlusion/viscosity sub-phenotypes associated with laboratory data in SCA. Legend:  (severe),  (mildest),  (severe),  (mildest),  (severe),  (mildest). T-Chol, HDL-C and LDL-C levels exhibit an association, suggesting a new dyslipidemic sub-phenotype. Lactate dehydrogenase (LDH) and indirect bilirubin are important biomarkers of hemolytic and endothelial dysfunction sub-phenotypes. Hematocrit (Ht), red blood cell (RBC) and MCV are associated with –α^3.7Kb^-thalassemia, reflecting the vaso-occlusive/viscosity sub-phenotype. However, we observed that reticulocyte count is associated with hemolysis and vaso-occlusion. HbF, NO metabolites and monocyte count show influence among all the proposed sub-phenotypes. The laboratory parameters described exhibit peculiar phenotypic diversity that was difficult for separating the pathophysiological mechanism of SCA

Considering our results, we corroborate with previously reports and described that these sub-phenotypes are dynamic, associated with several biomarkers alterations and can occur simultaneously in SCA, emphasizing the known associations concerning the pathophysiological processes present in the disease (Fig. 4).

## Conclusion

In accord to our data, LDH, NOm and HDL-C biomarkers were associated with the laboratory characterization, allowing the classification of hemolytic and dyslipidemic sub-phenotypes respectively. The co-inheritance of -α^3.7Kb^-thal and SCA improves the anemia and low platelet count; however, it contributes to increased blood viscosity. The laboratory parameters corroborate previously described hemolytic and vaso-occlusive/viscous sub-phenotypes. Our data are consistent with previous reports about dyslipidemia in SCA, thus we suggest the establishment of the dyslipidemic sub-phenotype. SCA has a peculiar phenotypic diversity that makes it difficult to determine individually which actually occurs simultaneously in the pathophysiology of the disease. Therefore, further studies should be developed in order to better understand the processes involved in association with systemic biomarkers of SCA.

## Abbreviations

ACS: Acute chest syndrome
Hb: Hemoglobin
*HBA*: Globin-α genes
HbF: Hemoglobin fetal
HDL-C: High-density lipoprotein cholesterol
Ht: Hematocrit
LDH: Lactate dehydrogenase
LDL-C: Low-density lipoprotein cholesterol
MCH: Mean corpuscular hemoglobin
MCV: Mean cell volume
NOm: Nitric oxide metabolites
RBC: Red blood cell
SCA: Sickle cell anemia
SCD: Sickle cell disease
VLDL-C: Very low-density lipoprotein cholesterol
VOE: Vaso occlusion events
-α^3.7Kb^-thal: -α^3.7Kb^-thalassemia
α-thal: α-thalassemia.
